# Long Non-Coding RNAs Regulating Immunity in Insects

**DOI:** 10.3390/ncrna3010014

**Published:** 2017-03-16

**Authors:** Valluri Satyavathi, Rupam Ghosh, Srividya Subramanian

**Affiliations:** 1 Centre of Excellence for Genetics and Genomics of Silkmoths, Centre for DNA Fingerprinting and Diagnostics, Hyderabad 500 001, India; srividyas@cdfd.org.in (S.S.); 2 Indian Institute of Science Education and Research, Bhopal 462023, India; rupam@iiserb.ac.in (R.G.)

**Keywords:** long non-coding RNAs, immunity, insects, silkworm

## Abstract

Recent advances in modern technology have led to the understanding that not all genetic information is coded into protein and that the genomes of each and every organism including insects produce non-coding RNAs that can control different biological processes. Among RNAs identified in the last decade, long non-coding RNAs (lncRNAs) represent a repertoire of a hidden layer of internal signals that can regulate gene expression in physiological, pathological, and immunological processes. Evidence shows the importance of lncRNAs in the regulation of host–pathogen interactions. In this review, an attempt has been made to view the role of lncRNAs regulating immune responses in insects.

## 1. Introduction

The success of insects in adverse environments indicates the advanced defense mechanisms employed by these organisms. With the passage of time, some insect species became resistant to most pathogens, and some remained susceptible to various infections. Insects exhibit both humoral and cellular immune responses against pathogens. The lack of an adaptive immune system has forced insects to choose immediate non-specific responses that include the production of antimicrobial peptides, phenoloxidase, apoptosis, phagocytosis, encapsulation, and nodulation. With the advent of molecular biology techniques, a large amount oftranscriptome data has been published and reviewed on the model organism *Drosophila melanogaster*, while other insect species have been investigated to a lesser extent. Transcriptome sequencing provides information on all transcripts occurring in different cells and tissues [1,2], while RNA sequencing provides data on multiple classes of non-coding RNAs (ncRNAs) [3,4,5,6].

Non-coding RNA represents a portion of RNA that does not code a protein, but contains information and remains functionally active by regulating other genes. Although 70% of the mammalian genome is transcribed, only a portion is used to produce proteins, and the rest produces non-coding RNA as final functional product. The non-coding RNAs derived from introns may be short (microRNAs) or long non-coding RNAs (lncRNA). The function of lncRNAs is assessed based on their low coding potential [5]. Although in vitro translation experiments with lncRNAs detected no formation of polypeptides, ribosome sequence analysis studies revealed that some annotated lncRNAs are involved in translation by elongating ribosomes. It was estimated that the coding potential of lncRNAs is low compared to that of coding RNAs. The evidence explains that many of the complex genetic interactions and variations between species are due to the regulatory role of non-coding RNAs, which seems to control gene expression during splicing, transcription, and/or translation.

Although there are a number of excellent reviews describing lncRNAs in various genomes [6,7,8,9,10], information on lncRNAs in insects is rather scattered. In insects, lncRNAs have been reported in *D. melanogaster* [11], *Anopheles gambiae* [12], *Apismellifera* [13,14], and *Bombyx mori* [15]. The present review is focused on the lncRNAs that are involved in immunity.

## 2. Identification and Classification of lncRNAs

The amount of noncoding genes varies among species, and only a small percentage of the genome represents protein coding genes. The ratio of non-coding to coding genes increases as the complexity of the organism increases. Earlier reports marked these “junk”regions as sponges for mutation and maintenance of the genetic material in both structural and regulatory manners. Recent findings suggest that ncRNAsdo contain information and regulate various levels of gene expression. The regulation of genes coding for proteins by non-coding genes mostly takes place at the transcriptional level (like enhancer sequences, alternative promoter sequences). However,even some of the non-coding genes may be transcribed efficiently but not translated (e.g. ribosomal RNA(rRNA), transfer RNA(tRNA). Though the importance of rRNA or tRNA has been known for ages, many other non-coding RNAs have been discovered, whose functions are still largely unknown.

This is because lncRNAs are mostly unconserved from species to species, and are expressed only in a space- and time-specific manner (e.g., expressed only in particular tissues or at particular developmental time points). lncRNAs are mostly classified based on their position relative to protein coding mRNAs like long intergenic lncRNAs, introniclncRNAs, antisense lncRNAs, transcribed pseudogene lncRNAs, and enhancer lncRNAs [16]. The lncRNAs can have either DNA binding sites, protein binding sites, or both, and can participate in variety of cascades. The databases dedicated to lncRNAs include LNCipedia and lncRNome [17,18], which describe their functions based on literature. NonCode [19] contains ncRNA sequences on a dozen model organisms, including *D. melanogaster*. It registers about 3193 lncRNAs from fruit fly predicted from RNASeq data, and does not provide any information on functional aspects. RNA Central repository [20] also has a lncRNA data base of a few insect models like fruit fly and honeybee.

In *D. melanogaster*, among various lncRNAs, the hswω- transcript was reported to form perinuclear omega-speckles in the nuclei in response to heat shock [21], *roX1*and*roX2* found in the male-specific lethal (MSL) protein complex affected dosage compensation [22], *yellow-achaete intergenic RNA*(*yar*) lncRNA influenced circadian rhythms [23], *CASK regulatory gene CRG* lncRNA regulated Ca^2+^/calmodulin-dependent protein kinase (CASK) transcription [24], and *Sphinx* lncRNA influenced courtship behavior [25].

In *Anopheles gambiae*, 2949 lncRNAs have been reported from multiple life stages using deep RNA-seq technology [12]. Jayakodi et al. identified 1514 intergenic lncRNAs (lincRNAs) in*Apismellifera*and 2470 lincRNAs in*Apiscerana*, and investigated their response to viral infection [13]. In*A. mellifera*, only six lncRNAs have been experimentally validated;four (*Nb-1*,*Ks-1*,*AncR-1*, and*kakusei*) of them were related to behavior [26,27,28], and the other two (*lncov1*and*lncov2*) were associated with ovary size [29].

## 3. lncRNAs in Mammalian Immune Response

Recent evidence indicates that lncRNAs have an important regulatory role in immunity and host–pathogen interactions. Zhang andCao [30] reviewed the role of lncRNAs in development and immune responses through different mechanisms, such as dosage compensation, imprinting, enhancer function, transcriptional regulation, and post-transcriptional regulation. Although this review’s main focus is on insects, due to the existence of few reports on lncRNAs in insects, a couple of mammalian examples wherein lncRNAs are reported in the regulation of immune responses arediscussed.

The Guttman [31] group was the first to report a role for lncRNAs in innate immunity. Using information from RNA-seq analysis, the group reported differential expression of lncRNAs upon the activation of monocytes, macrophages, dendritic cells, and fibroblasts in mammals. Most of the lncRNAs—e.g.,THRIL (TNFα and hnRNPL related immunoregulatory lincRNA), PACER (p50 associated COX-2 extragenic RNA), lnc-IL7R, and IL1β-RBT46 [8,9,32,33,34]—were reported to regulate immune responses in *cis*, while many other lncRNAs function in *trans*. Examples where lncRNAs can target immune responses are receptors and the transcription factors nuclear factor kappa B (NFκB) andSTAT3,whichregulate Toll, IMD, and JAK STAT signaling pathways.

In case of mice, Severe Acute Respiratory Syndrome (SARS) coronavirus infection of the lungs resulted in differential expression of lncRNAs [35]. Additionally, lncRNAs were reported to express following lipopolysaccharide (LPS) stimulation in mouse macrophages [10]. Following Pam3CSK stimulation, an lncRNA named lincRNA-COX2 was reported to regulate about 1500 genes in mouse macrophages [36].

*Lethe* isa pseudogene lncRNA activated by tumor necrosis factor (TNF) and interleukin1 beta (IL1β) was reported to negatively regulate nuclear factor kappa B, and is linked directly to the control of inflammatory response [37]. Toll-like receptor signaling, which targets a variety of immune related genes, also induces lincRNA-COX2, which interacts with heterogeneous nuclear ribonucleoproteins [38].

NEAT1 expression is induced after Poly (IC) or influenza stimulation in HeLa cells, which triggers the redistribution of SFPQ and increased CXCL8 expression [39]. Ptprj-as1, IL1β-RBT46, and IL-1β-enhancer RNA (eRNA) have been shown to regulate IL1β and CXCL8 expression when stimulated with LPS [12,40]. Long non-coding dendritic cell (lncDC) was reported to be essential for the differentiation of monocytes into dendritic cells and triggering STAT3 [41]. In mice, infection due to Theiler’s Virus and *Salmonella* was controlled by lncRNA NeST by epigenetic regulation of interferon gamma IFNγ locus [42,43].

## 4. lncRNAs in Insect Immunity

In case of insects, lncRNAs have been characterized only in few species like *Drosophila*, *Apismellifera*, and *Bombyx mori*. Table 1 provides a list of lncRNAs identified in insects. The lncRNAs are usually classified as those involved in development, behavior, or neural expression. Although lncRNAs are reported in insects like *Aedesgambiae*, *Anopheles gambiae*, *Danausplexippus*, or *Heliconiusmelpomene*, no in-depth functional analysis is available. In *Triboliumcastaneum*, numerous lncRNAs were reported to express on the antisense strand of protein-coding genes localized in the Hox cluster [44]. In the case of *Nasoniavitripennis*, a vast study on the transcriptome of testis tissue revealed the presence of four putative ncRNAs [45]. In silkworm, deep transcriptome sequencing of 18 different tissues combined with public RNA-seq datasets of three silkworm tissues revealed lncRNAs with low expression levels, high spatial specificity, and low sequence conservation [46]. A proportion of lncRNAs were reported to be involved in the biosynthesis, translocation, and secretion of silk proteins. Only one lncRNA *Fben-1* was reported from transcriptome sequencing of brain tissues of female moth collected from fifth instar silkworm larvae [46,47]. Li et al. observed that some lncRNAs are transcribed from the silk gland of *B. mori*, and reported their involvement in the repression of transcription by the epigenetic modification of histones [48].

Several lncRNAs were found to be selectively expressed in the domesticated silkworm *B. mori* upon *Bombyx mori* Nucleopolyhedrovirus (BmNPV) infection, but little is known about their functional role. We have identified 1173 putative lncRNAsfrom a total of 11,160 full-length cDNAs (KAIKObase) based on the criteria that the sequences had no exonic overlap in sense with reported protein coding genes. About 37 such sequences were tested for their protein coding potential. Based on coding potential and differential expression pattern in thecomplementary DNA(cDNA) library derived from midgut and fat body tissues of BmNPV infected fifth instar larvae of resistant (SBNP1) and susceptible (CSR2) silkworm strains, four putative lncRNA were selected for further investigation. Time course analysis revealed differential expression of lncRNAs in the midgut and fat body tissues in the resistant and susceptible strains of *B. mori.*Out of four lncRNAs, lncRNA4 (scaffold nscaf2674 and sequence length 1,473,305–1,473,715) with a difference in FPKM (Fragments Per Kilobase of transcript per Million mapped reads) as 4 and −1.33 coding potential was identified to express differentially (410 bp) only in the susceptible CSR2 strain [56]. lncRNA 4 showed high expression at 48 and 96 h post infection both in the fat body and midgut tissues of the infected *B. mori* larvae.

## 5. Mode of Action

**Figure 1:**
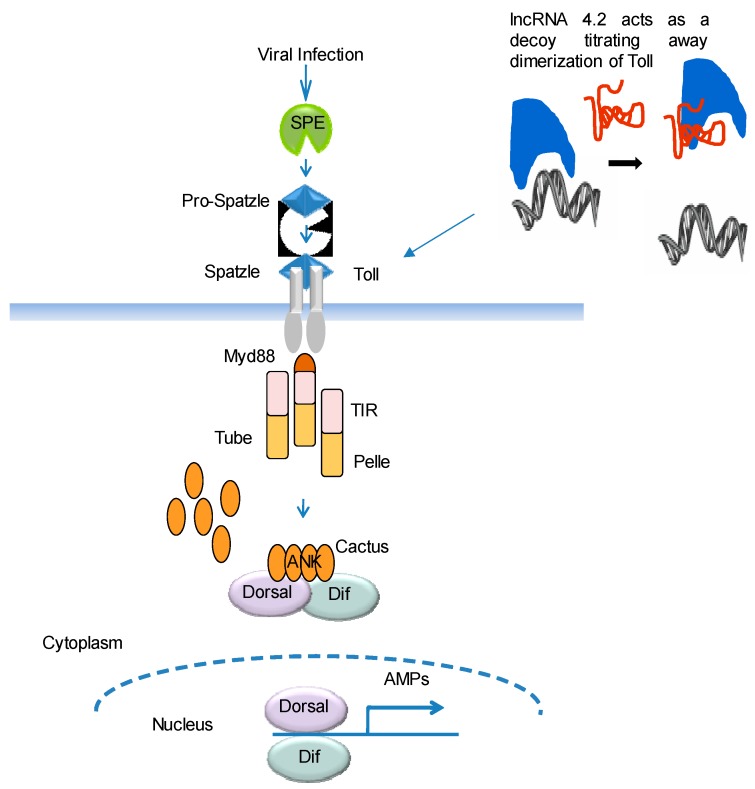
Schematic representation of long non-coding RNA (lncRNA) regulation of immune response pathway. It is hypothesized that lncRNAs can act as a decoy and titrates away dimerization of Toll on the plasma membrane, resulting in dysregulation of the Toll signaling pathway. TIR-Toll/Il-1 receptor domain, SPE – spätzle processing enzyme, ANK- ankyrin repeat domain, AMP- antimicrobial peptides, Dif-nuclear factor-kB like protein.

The mode of action of the lncRNAs in immunity has so far not been elucidated in any insect. lncRNAs have been reported to regulate biological processes during development or following a stress by means of epigenetic control of chromatin (re)organization or RNA sequestration in a nuclear compartment and/or neighboring cis-regulation of specific mRNA genes.In the case of *B. mori*, we identified lncRNA4, whichshowed involvement in Tollsignaling. The comparative studies on the expression of levels of lncRNAs and immune genes (*Tolls*) revealed that lncRNA4 followed a similar expression pattern comparable to that of *Toll4*. We speculate that lncRNA4 might be acting as a decoy and titrates away the dimerization of Toll on the membrane, preventing activation of *Toll.* Figure 1 depicts regulation of immune response pathway by lncRNA. In general, dimerization of Toll is required for phosphorylation of *Cactus* and transport of *rel* factors *Dorsal* and *Dif* to the nucleus to produce antimicrobial peptides.

The knowledge in the field of lncRNAs, and their mode of action and function can provide deep insight into the evolution and function of genomes during host–pathogen interactions.

## 6. Conclusions

Studies in the host–pathogen interaction have mostly been restricted to the protein coding genes. Although huge in size, thenon-coding regions of the genome are not investigated. Recent studies are opening a new layer of regulation of cellular processes, including immune response by non-coding RNAs. Current studies indicate that the present knowledge is only the tip of the iceberg, and much research is needed in this field to solve the non-coding enigma. The identification of lncRNAs continues to pose a challenge due to a lack of evolutionary conservation as well as lack of protein product, which hinders the development of a good algorithm for its annotation. Despite these limitations, many lncRNAs have been studied and a mode of regulation has been proposed which awaits early attention from researchers. Proper understanding of host–pathogen interaction is essential to decode the intricacies of the immune mechanisms employed by organisms against various pathogens, especially viruses. Delving into the depth of mechanisms will allow us to provide a simple model system to understand the antiviral mechanism of higher eukaryotes.

## Figures and Tables

**Table 1:** lncRNAs reported in insects.

Species	Gene	Function	Reference
*Anopheles gambiae*	*RNAseq*		
*Apis mellifera*	*AncR-1*	Neural expression	[27]
	*Kakusei*	RNA metabolism	[14]
	*Ks-1*	Neural expression	[28]
	*Lnccov1/2*	Autophagic cell death of ovarioles	[29]
	*Nb-1*	Putative role in polyethism	[49]
*Bombyx mori*	*Fben-1*	Biosynthesis, translocation, and secretion of silk proteins	[46,47]
*Drosophila melanogaster*	*bithora*	Development of abdominal segments	[50]
	*hsr-w*	Heat shock stress	[21]
	*roX1/2*	Dosage compensation	[22]
	*sphinx*	Regulates sensory circuits	[25]
	*yar*	Regulator of yellow and achaete transcription	[23]
*Plutella xylostella*	*lincRNA*	Detoxification and toxin related metabolism	[51]
*Plasmodium falciparum*	*lncRNA-TARE*	Plays a role intranscriptional regulation	[52]
	*Var*	Regulates var gene activation	[53]
	*PAN*	Facilitates switch from latent to lytic infection	[54]
*Spodoptera frugiperda*	*LNCR*	Formation of heterochromatin	[55]
